# Supplementary material for: Solar Powered Microplasma-Generated Ozone: Assessment of a Novel Point-of-Use Drinking Water Treatment Method
Source: Int J Environ Res Public Health. 2020 Mar 13;17(6):1858. doi: 10.3390/ijerph17061858 (PMC7175310; doi:10.3390/ijerph17061858)
Supplement: Supplementary file 1 [file ijerph-17-01858-s001.pdf]

Supplemental Information

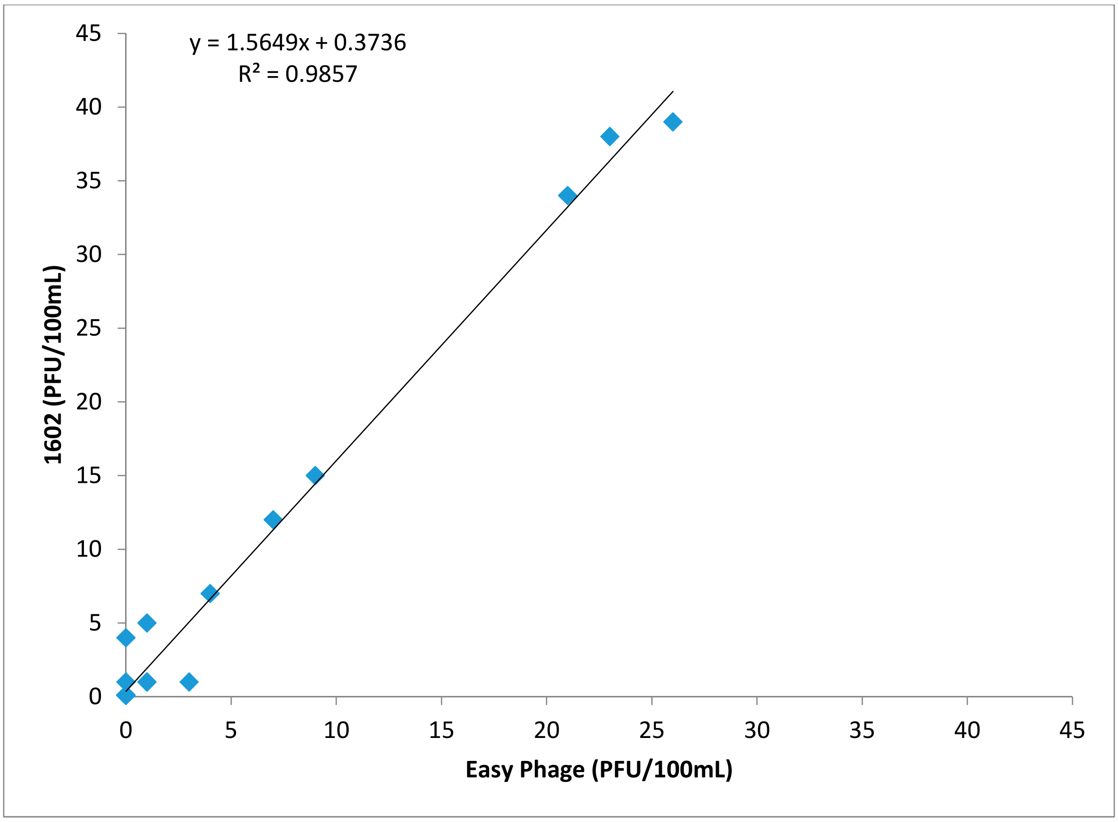

Supplemental Information Figure S1: Association between F+ coliphage measured using a simplified rapid method, and EPA method 1602.

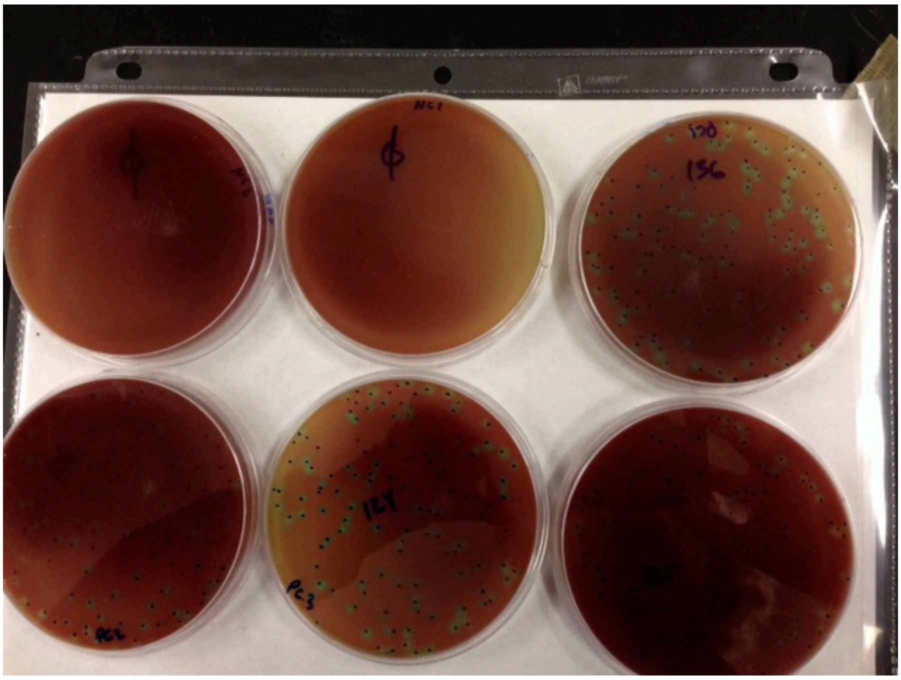

Supplemental Information Figure S2: Appearance of EasyPhage plates after 19 hours of incubation with positive (MS2) controls.

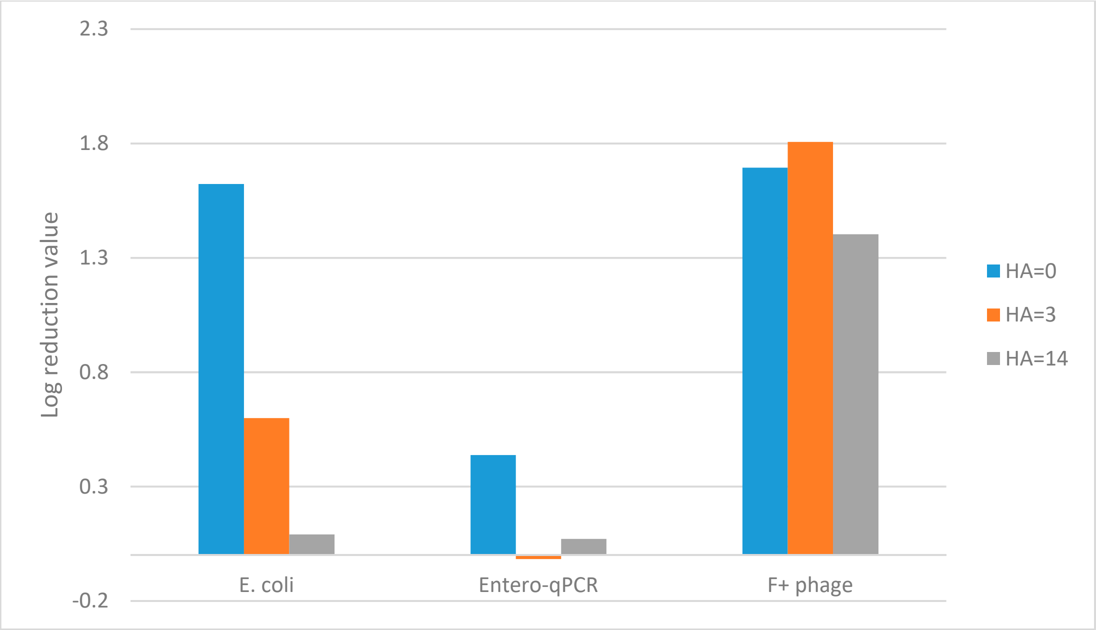

Supplemental Information Figure S3: Impact of humic acid on the reduction of indicator microbes at time=60 minutes, by ozonation. HA= indicates the mass of humic acid (in grams) added to 100L of treated water.
